# Single-Cell and Single-Cycle Analysis of HIV-1 Replication

**DOI:** 10.1371/journal.ppat.1004961

**Published:** 2015-06-18

**Authors:** Mowgli Holmes, Fengwen Zhang, Paul D. Bieniasz

**Affiliations:** 1 Columbia University, New York, New York, United States of America; 2 Aaron Diamond AIDS Research Center and Laboratory of Retrovirology, The Rockefeller University, New York, New York, United States of America; 3 Howard Hughes Medical Institute, New York, New York, United States of America; Universitätklinikum Heidelberg, GERMANY

## Abstract

The dynamics of the late stages of the HIV-1 life cycle are poorly documented. Viral replication dynamics are typically measured in populations of infected cells, but asynchrony that is introduced during the early steps of HIV-1 replication complicates the measurement of the progression of subsequent steps and can mask replication dynamics and their variation in individual infected cells. We established microscopy-based methods to dynamically measure HIV-1-encoded reporter gene and antiviral gene expression in individual infected cells. We coupled these measurements with conventional analyses to quantify delays in the HIV-1 replication cycle imposed by the biphasic nature of HIV-1 gene expression and by the assembly-inhibiting property of the matrix domain of Gag. We further related the dynamics of restriction factor (APOBEC3G) removal to the dynamics of HIV-1 replication in individual cells. These studies provide a timeline for key events in the HIV-1 replication cycle, and reveal that the interval between the onset of early and late HIV-1 gene expression is only ~3h, but matrix causes a ~6–12h delay in the generation of extracellular virions. Interestingly, matrix delays particle assembly to a time at which APOBEC3G has largely been removed from the cell. Thus, a need to prepare infected cells to be efficient producers of infectious HIV-1 may provide an impetus for programmed delays in HIV-1 virion genesis. Our findings also emphasize the significant heterogeneity in the length of the HIV-1 replication cycle in homogenous cell populations and suggest that a typical infected cell generates new virions for only a few hours at the end of a 48h lifespan. Therefore, small changes in the lifespan of infected cells might have a large effect on viral yield in a single cycle and the overall clinical course in infected individuals.

## Author Summary

The HIV-1 replication cycle is composed of several sequential steps. While the timing of the early steps of HIV-1 replication is quite well understood, measuring the duration of later steps is complicated by the fact that asynchrony is introduced into populations of infected cells during early steps. We devised imaging methods for measuring the duration of late steps in HIV-1 replication in single infected cells, circumventing the problems associated with measurements in populations of asynchronously infected cells. By combining these measurements with conventional analyses of HIV-1 replication in populations of cells, we derived a time-line of key events during the late steps of the HIV-1 life cycle. We find that the delay between early and late gene expression is small but that a subsequent programmed delay in virus assembly enables HIV-1 to remove a host antiviral protein from infected cells before new virions are generated. In so doing, HIV-1 may prevent futile virion production.

## Introduction

The HIV-1 replication cycle consists of several discrete, sequentially occurring processes, involving numerous viral and host cell components. For the early steps in HIV-1 replication, there is a reasonably good appreciation of the kinetics with which individual steps occur. Viral entry occurs over about <1–3h after exposure of cells to virus [1–5], reverse transcription occurs over the ensuing 6 to 48 hours [5–10] and integration takes place about 5 hours after the completion of reverse transcription [6, 7]. The dynamics of the early steps in HIV-1 replication, particularly entry and reverse transcription, appear to be cell type dependent, and the rather large variability in published estimates of these dynamics may be due to variation in receptor and intracellular dNTP levels.

In contrast, the dynamics of the various steps of the post-integration phases of the viral life cycle, e.g. the relative timing of the onset of early versus late gene expression, and the timing of particle assembly/release relative to viral gene expression are comparatively poorly documented. A challenge in determining the dynamics of HIV-1 replication is its inherent asynchrony in populations of cells, which can obscure the underlying dynamics in individual cells. Nevertheless, time-of-addition experiments indicate that resistance to transcription inhibitors is acquired at ~35h after infection [11, 12].

Interestingly, the overall time taken to complete an HIV-1 replication cycle is broadly similar to the lifespan of an infected cell in its natural environment [13]. Approaches in which mathematical modeling was coupled with measurements of viral RNA in the blood of patients beginning anti-retroviral treatment or undergoing plasmapheresis have provided estimates for the lifetime of infected cells and the generation time of HIV-1 in a natural setting [14–18]. Successive refinements of these models led to the conclusions that that the viral generation time and the average lifetime of infected cells were about 48 hr [13].

The fact that the lifespan of an infected cell *in vivo* is somewhat similar to time taken to complete an HIV-1 life cycle suggests the lifespan of the cell should limit burst size. Given that cells are at risk of death through cytopathic effects and/or lysis from cytotoxic immune cells from the time at which they begin to express viral proteins, one might expect viral variants to be selected that minimize the time interval between the onset of viral protein expression and release of infectious virions. In other words, there should exist a selection pressure to optimize each of the late (post transcriptional) phases of viral replication cycle for speed [19]. It is intuitively surprising, therefore, that at least two features of the HIV-1 replication cycle appear to extend the time interval between onset of viral protein expression and the generation of infectious viral particles. First, to express nine genes from a highly compact genome with a single promoter, HIV-1 employs extensive alternative splicing, with unspliced, singly spliced, and multiply-spliced mRNAs providing all the necessary templates for translation of viral proteins [20]. Because incompletely spliced mRNAs do not efficiently exit the nucleus, the so-called ‘early’ HIV-1 proteins are products of fully spliced mRNAs. The viral Rev protein is one such early protein that shuttles between the nucleus and the cytoplasm, moving intron-containing viral mRNAs from nucleus to cytoplasm [20–25] via the CRM1 nuclear export pathway [26–29]. The Rev-dependence of ‘late’ genes (Gag, Pol, Env, Vif, Vpr and Vpu) predicts that their expression should be delayed so that viral gene expression is divided temporally into two phases. However, there is a paucity of data on the dynamic structure of early versus late gene expression during HIV-1 replication in the context of a 48h replication cycle. Early studies of mRNA production using northern blot or semi-quantitative PCR analyses of populations of infected cells [25, 30] suggest that late, incompletely spliced transcripts are expressed ~0 to 12h after early completely spliced transcripts. Thus, the magnitude and significance of the delay in the replication cycle imposed by the biphasic nature of HIV-1 gene expression is unclear.

Another delay in the late stages of the HIV-1 replication may occur during particle assembly. The binding of Gag to cell membranes, a necessary step in particle assembly, is driven by the N-terminal matrix (MA) domain of Gag, via a basic patch and a myristate group linked to the N-terminal glycine residue [31, 32]. However, MA-membrane binding is inhibited by cellular tRNAs [33, 34] and via a myristoyl-switch mechanism in which the N-terminal myristate is sequestered in a hydrophobic pocket in the MA globular head when Gag is monomeric [35–37]. Thus, mutations in MA or, more dramatically, deletion of the MA globular head, can induce increased membrane binding and increased levels of virion release [38–40], particularly at the low Gag concentrations at which WT Gag is usually cytoplasmic [41, 42]. Since Gag concentration in infected cells is expected to be low initially, and to progressively increase with time, these findings suggest that HIV-1 Gag actively delays its own assembly into particles. Because this property should lengthen the viral replication cycle, there are likely to be important functional reasons why virion assembly is inhibited or delayed in the context of wild-type HIV-1.

A possible impetus for the acquisition of programmed delays in the HIV-1 life cycle may be the apparent need for HIV-1 to manipulate the cellular environment in advance of virion generation. In particular, cells express a number of molecules that can inhibit viral replication [43, 44]. To allow particle assembly to proceed prematurely in the presence of high levels of such molecules may be futile, or even deleterious to overall virus replication. For example, APOBEC3G (A3G) is a constitutively expressed antiretroviral protein that is packaged into virions and catalyzes lethal hypermutation of nascent viral DNA during reverse transcription [45]. However, human A3G is bound by the HIV-1 accessory protein Vif, a late gene product, that recruits a ubiquitin ligase complex to drive A3G degradation [45–48]. The timing of the removal or downregulation of A3G, and other proteins that exert antiviral activity, relative to viral replication is unknown, but A3G depletion must follow late gene expression.

In this study we attempted to quantify (i) The delay in HIV-1 replication imposed by the biphasic nature of HIV-1 gene expression, (ii) the delay in particle assembly that is attributable to MA and (iii) the dynamics of restriction factor removal (using A3G as a specific example) relative to viral gene expression and particle release. To ameliorate the difficulties associated with measuring dynamics in asynchronously infected cells, we utilized a combination of techniques, including quantitative fluorescence microscopy of single cells wherever possible. We supplemented these studies with conventional analyses of virion production. These studies provide a timeline for key events in the HIV-1 replication cycle and reveal that the interval between the onset of early and late HIV-1 gene expression is only ~3h, but the globular head of MA causes an additional ~6–12h delay in virion assembly. Interestingly, the globular head of MA delays particle assembly to beyond the time at which A3G has largely been removed from the cell. Thus, the need to remove inhibitory molecules, and otherwise prepare the cell to be an efficient producer of virions, may provide an explanation for the apparent requirement that HIV-1 actively inhibits the pace of particle assembly.

## Results

### Dynamics of early and late gene expression in populations of HIV-1 infected cells

**Fig 1:**
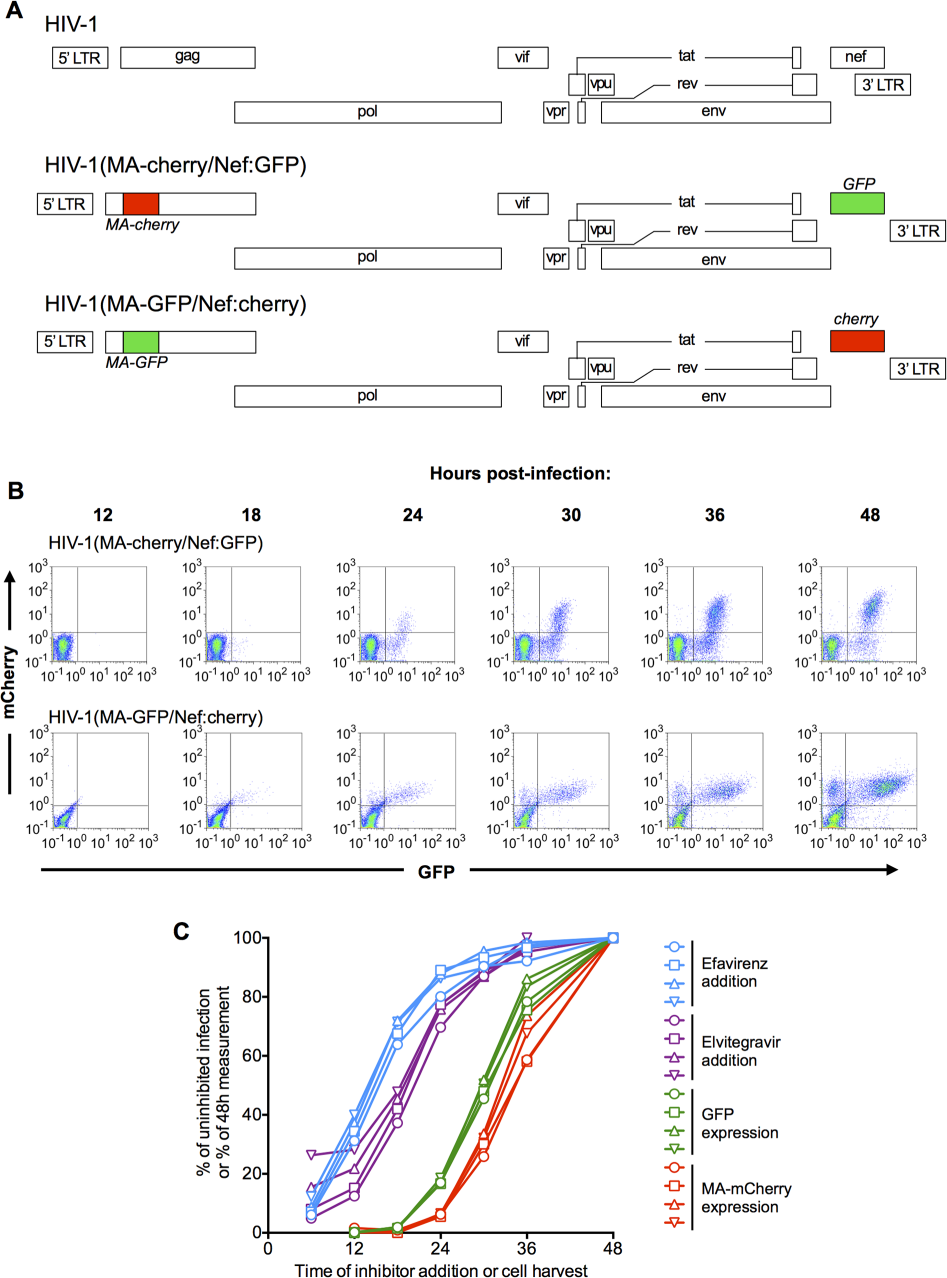
Dynamics of HIV-1 replication measured using dual reporter viruses. (A) Schematic representation of the HIV-1 genome, and derivatives HIV-1(MA-cherry/Nef:GFP) and HIV-1(MA-GFP/Nef:cherry) encoding dual-reporters to study early and late gene expression. (B) Flow cytometric analysis of MT4 cells following synchronized infection with each of the dual-reporter viral constructs displayed in (A). FACS plots with gates for GFP-positive and mCherry-positive cells are displayed. (C) MT4 cells were infected with HIV-1(MA-cherry/Nef:GFP) at four different MOIs: 0.1 (circles), 0.2 (squares), 0.4 (triangles), and 0.8 (inverted triangles). Inhibitors of reverse transcription (Efavirenz, blue lines and symbols) or integration (Elvitegravir, purple lines and symbols) were added at the indicated times. The cells were fixed at 48h. post-infection and analyzed by flow cytometry, and the number of infected cells (as indicated by expression of a Nef:GFP reporter) is plotted as a percentage of that observed if inhibitor was never added. Alternatively, cells were harvested at the indicated times and the number of GFP-positive and mCherry positive cells plotted as a percentage of the 48h maximum value.

To determine the relative timing of the early and late phases of HIV-1 gene expression, we constructed proviral plasmids encoding fluorescent proteins in positions that would be expressed via a completely spliced early transcript or via an unspliced Rev-dependent late transcript (Fig 1A). The early-gene reporter was inserted in place of *nef*, which is expressed at high levels compared to the other early genes [20] but is not required for viral gene expression or replication. The late gene reporter was inserted near the C-teminus of MA in the Gag polyprotein, at a site known to tolerate such insertions [49]. One reporter construct, termed HIV-1 (MA-cherry/Nef:GFP), had GFP in the early gene position and mCherry in the late gene position, while another, termed HIV-1(MA-GFP/Nef:cherry) carried the reporters in exchanged positions (Fig 1A). By employing both constructs in these assays we could ensure that differences in the measured kinetics of early versus late gene expression were not artifacts of differential fluorophore intensities or maturation rates. The viral reporter genomes contained inactivating mutations in the catalytic sites of the reverse transcriptase and integrase enzymes, and a deletion in Env. Thus, replication was restricted to a single cycle, and reporter genomes were introduced into cells using virions generated by co-transfection of the reporter genomes with HIV-1 GagPol and VSV-G expression plasmids.

To track the dynamics of early and late gene expression, we infected MT4 cells as synchronously as possible. This was done by spinoculating cells at 15°C, briefly incubating them at 37°C (90 min), and then removing unattached virions by washing. Expression of the reporter genes was first detected at 18–24h after infection, and the numbers of cells expressing the early gene reporter alone, or expressing both reporters increased progressively thereafter (Fig 1B). As expected, given that late gene expression is dependent on early gene products, the percentage of cells expressing the early gene reporter was always higher than that expressing the late gene reporter and virtually no cells expressed the late reporter without also expressing the early reporter (Fig 1B). An unanticipated observation was that both GFP and mCherry reporters were expressed at higher and more heterogeneous levels when they were expressed using a late gene transcript than via the early gene transcript (Fig 1B). However, this finding is consistent with previous analyses of viral mRNA levels in infected cell populations [25]. We compared the dynamics of early and late reporter gene expression in MT4 cells with those in primary T-cells (S1 Fig). While the dynamics of reporter gene expression in primary cells varied somewhat with individual donor, and more so with how the cells were activated (S1A–S1I Fig) the times after infection at which expression of the early and late reporter genes were detected in cell lines (MT4 and HOS, S1J–S1L Fig) was quite similar to that in primary T-cells. Therefore, unless otherwise stated we used MT4 cells for convenience and reproducibility thereafter.

To place the timing of the late steps of HIV-1 replication in context, we derived an estimate of the timing of early events in the replication cycle in our MT4 cell experimental system. To accomplish this, we first synchronously infected cells with one of the dual reporter vectors (HIV-1 (MA-Cherry/Nef:GFP)). Thereafter, reverse transcription and integration inhibitors were applied at varying time points and the fraction of infection events that were no longer susceptible to the inhibitors at each time of inhibitor addition was determined (Fig 1C). To increase the robustness of this analysis, infections were done at 4 different multiplicities of infection (MOI). We fitted 5-parameter sigmoid functions to these each of these 4 data sets and calculated the times at which each curve reached 50% of its maximum value. This analysis indicated that half of the incoming virions had completed reverse transcription at 14.4±0.4h and integration at 19.3±1.1h post-inoculation (Fig 1C).

To obtain an estimate of the timing of the onset of early and late gene HIV-1 expression in populations of cells, we again infected cells at the 4 different MOI and used FACS to determine the fraction of cells that expressed detectable levels of early or late reporter genes at various times after infection (Fig 1C). Fitting 5-parameter sigmoid functions to these 4 data sets demonstrated that half of the infected cells expressed detectable levels of the early gene reporter at 30.1±0.4h and detectable levels of the late gene reporter at 33.4±0.4h after infection (Fig 1C). Thus, the interval between integration and detection of early gene expression estimated by these methods was 10.8h, while the interval between early and late gene expression was 3.3h, or ~10% of the duration of the viral replication cycle up to that point.

### Measurement of early and late HIV-1 gene expression in individual cells

**Fig 2:**
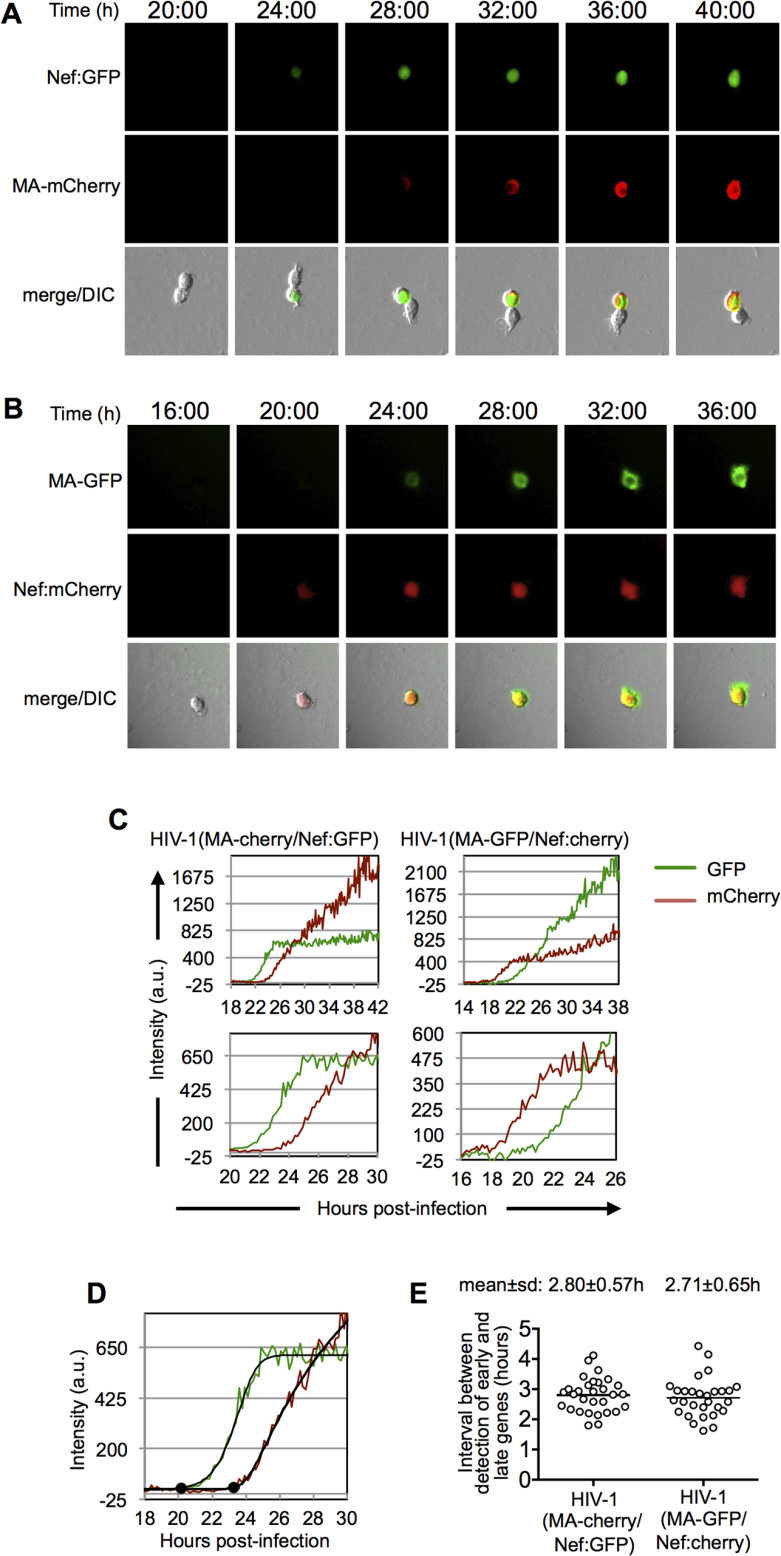
Dynamics of early and late gene expression in individual cells. (A and B) Examples of images of individual MT-4 cells infected with HIV-1(MA-cherry/Nef:GFP) (A) and HIV-1(MA-GFP/Nef:cherry) (B) dual-reporter virus. The time post-infection (in hours) is indicated at the top of the image sets. (C) Examples of quantitation of fluorescent intensities for individual cells infected with HIV-1(MA-cherry/Nef:GFP) and HIV-1(MA-GFP/Nef:cherry) dual-reporter viruses. Intensity units are 12-bit grey-level values, from 0–4095. Upper panels show data from entire experiments, lower panels show a magnified portion of the data around the time that early and late gene expression became detectable. (D) Example of a 5-parameter logistic curve fit to the raw data and time points at which early and late gene expression is detected, defined here as the first point at which the fit-curve rises 7 intensity units above its lower bound. The black dots indicate the identified time points for the detection of early and late gene expression. (E) Time interval between the detection of early and late gene expression, as defined in (D) for 30 individual HIV-1(MA-cherry/Nef:GFP) infected cells and 29 individual HIV-1(MA-GFP/Nef:cherry) infected cells. Fluorescence data for each of these individual cells is shown in S2 and S3 Figs.

The FACS-based methods described above analyzed gene expression in individual cells, but were based on counting the number of cells in a population whose fluorescence had crossed a threshold detection value. Thus, FACS analyses could not provide information about how the fluorescence of individual cells changes over time, or how the interval between the onset of early and late gene expression might vary among individual cells in a population. For this reason, and to provide a second method for estimating the interval between early and late gene expression, we established a microscopy method to dynamically measure reporter gene expression in individual cells. Following exposure to the reporter virus inoculum, infected MT4 cells were washed and immobilized. Images of cells were collected at 2–5 min intervals from ~12h to ~48h following infection (Fig 2A and 2B and S1–S4 Movies), and fluorescence intensity associated with the early- and late-gene reporters quantified (Fig 2C).

While, as expected, there was variability in the timing and level of reporter expression among individual infected cells, their overall behavior was stereotypic (Fig 2A and 2B and S1–S4 Movies). Specifically, the early gene reporter became detectable in individual cells ~14 to 30h after infection, and the late gene reporter became detectable shortly thereafter, 1.3 to 4.7h later. Both early-gene and late-gene reporters exhibited a rapid increase in intensity for the first several hours after they became detectable. During this phase, the levels of the early and late reporters increased at similar rates, so that the trajectories of the fluorescent intensity measurements were approximately parallel (Fig 2C). A few hours after the time at which early-gene reporter became detectable, there was a clear change in the trajectory of early gene expression, after which the levels of the early gene reporter plateaued, or increased less rapidly. The late gene reporter did not show this behavior. Rather, the levels of late reporter increased for the duration of the experiment, with only marginal tendency for the rate of increase to slow toward the end of a 40–48h imaging experiment (Fig 2C). This finding is concordant with the behavior of populations of cells analyzed by flow cytometry (Fig 1B), in which the fluorescence intensity associated with the late reporter increased over time, ultimately reaching higher levels than the early gene reporter.

We used the microscope-based assay to quantitate the interval between early and late gene expression in individual cells. To accomplish this in an unbiased manner, we used a MATLAB script to fit a 5-parameter logistic equation to the relevant regions of each of the fluorescent intensity plots to generate sigmoid best-fit curves, and to identify the points at which fluorescence rose above background (Fig 2D). Background fluorescence was measured in an unoccupied control region close to each cell and subtracted from the cell-associated fluorescence. The resulting fluorescence intensity data had initial levels that were close to zero, corresponding to the lower bound of the best-fit curve. The time at which the intensity curves became detectable above background, was defined as the time at which the fitted curve was at least 7 intensity units above its horizontal lower bound (Fig 2D).

Using this approach we found that the mean±s.d. interval between the initiation of HIV-1 early and late gene expression was 2.8±0.6h with a range of 1.6 to 4.4h in 59 individual infected cells (S2 and S3 and 2D Figs). Importantly, this result was not significantly affected by the identity of the fluorophore assigned to the early and late gene reporter positions (S2 and S3 and 2D Figs). Although there was substantial variation in the interval between early and late gene expression among individual infected cells, the mean value was in reasonable agreement with the value of 3.4h obtained using FACS analysis of populations of cells (Fig 1C).

### Kinetics of HIV-1 particle generation

To determine the timing of HIV-1 assembly during a single cycle of HIV-1 replication, we modified a replication-competent viral clone (NHG) that carries a single reporter gene (GFP) in the Nef position. An NHG derivative lacking the globular head of matrix (ΔMA) was used to quantify the effect of the globular head of MA. VSV-G pseudotyped virions were used to infect MT-4 cells, leading to the production of virions that were incapable of initiating a second round of infection in the culture.

**Fig 3:**
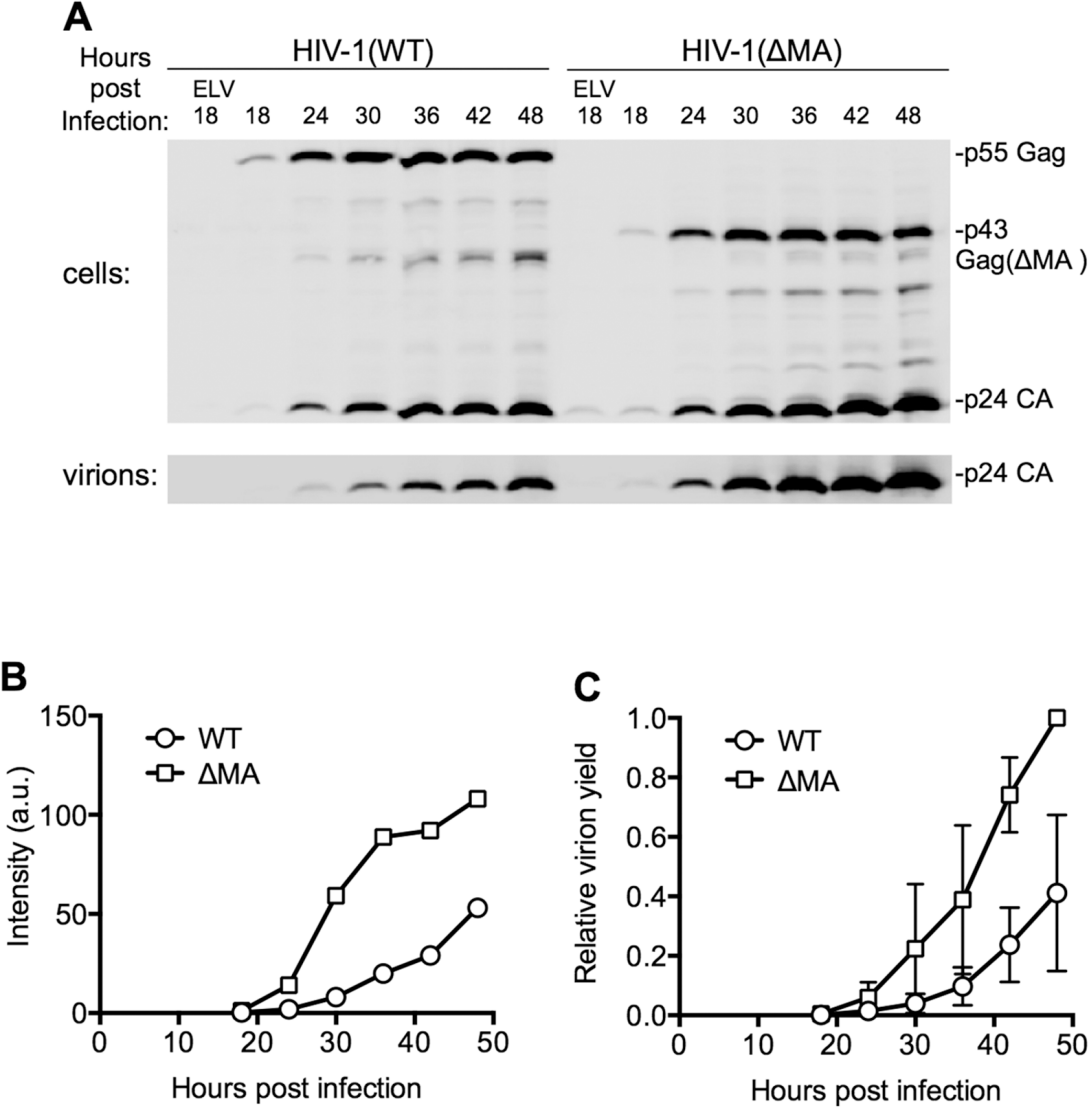
Effect of the MA globular head on the dynamics of HIV-1 replication. (A) Representative Western blot (anti-CA) analysis of cell and virion associated Gag following infection with HIV-1(WT) and HIV-1(ΔMA) at an MOI of 0.7. Cells and virons were harvested at the indicated times after infection. ELV, Elvitegravir, an integrase-inhibitor was added to some samples to block infection to indicate background levels of CA due to input virus. (B) Quantitation of the intensity of the virion-associated CA from the experiment shown in (A), using LiCOR Odyssey scanner, and assigned arbitrary units (a.u.). (C) Quantitation of the intensity of the virion-associated CA following infection on MT4 cells with HIV-1(WT) and HIV-1(ΔMA) at an MOI of 0.2 to 1. Values were normalized to the maximum level of virus release in each experiment, that was arbitrarily assigned a value of 1. The mean ± sd (n = 4 to 7) for each time point is plotted.

Although it was not possible to measure virion release by individual cells, the amount of viral protein associated with virions released into the culture supernatant by populations of cells could be measured using western blot assays (Fig 3A). These analyses both showed that the ΔMA virus indeed generated extracellular virions more rapidly and in greater quantities than WT virus (Fig 3A and 3B and 3C). These measurements were quite variable (Fig 3C), and MOI-dependent, presumably because the efficiency of virion assembly is dependent on Gag expression level, particularly in the case of the WT virus. Nevertheless, using the time point at which the yield of extracellular WT virus had reached its maximum level (at the end of the 48h experiment) as a reference point, we found that the ΔMA virus achieved this level of extracellular virions 12.5±5.4h (n = 7) earlier than WT virus (Fig 3C). Moreover, the total deficit in virion production over the course of a 48h experiment that could be ascribed to MA was 4.4±3.2 fold (n = 7). Thus, there was clear penalty in replication kinetics and overall particle yield imposed by the globular head of MA.

### Dynamics of A3G downregulation in populations of HIV-1 infected cells

To determine the dynamics of A3G depletion from infected cells relative to HIV-1 gene expression, we constructed two cell lines, based on MT-4 and HOS, stably expressing an mCherry-A3G fusion protein. These cells were infected with HIV-1(Nef:GFP) or HIV-1(MA-GFP) carrying a GFP reporter as an early or late gene. Thus, cells became green fluorescent as HIV-1 early or late gene expression commenced, and they lost their red fluorescence as mCherry-A3G was degraded through the action of Vif.

**Fig 4:**
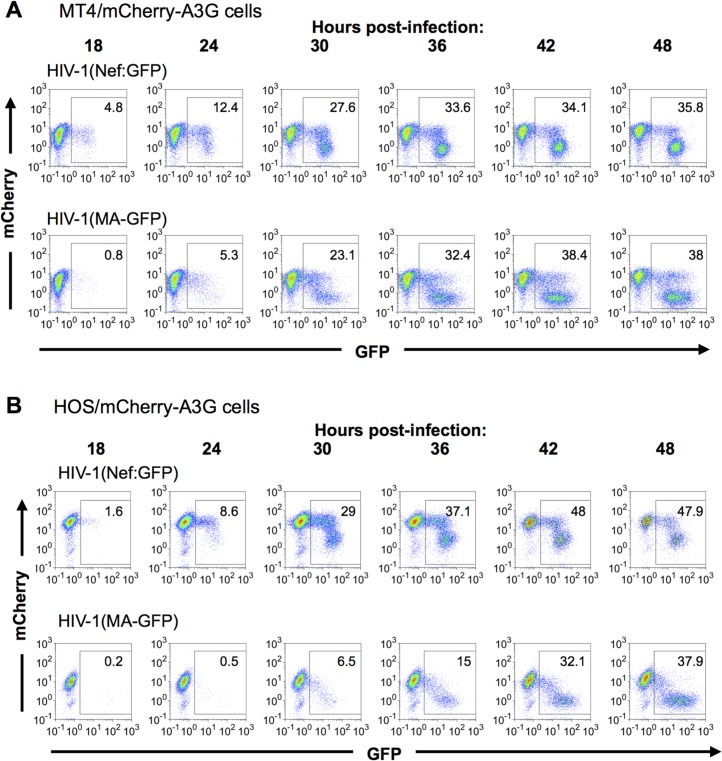
Dynamics of A3G down-regulation in populations of HIV-1 infected cells. (A, B) MT-4 cells (A) or HOS cells (B) stably expressing an mCherry-A3G fusion protein were infected with HIV-1(Nef:GFP) (upper rows) or HIV-1(MA-GFP) (lower rows). Cells were collected and fixed at the indicated time points, and levels of GFP and mCherry fluorescence analyzed by flow cytometry.

We first determined the kinetics of A3G removal relative to HIV-1 gene expression in populations of cells, using FACS analyses (Fig 4A and 4B). In mCherry-A3G expressing cells infected with HIV-1 carrying a GFP reporter in either early or late gene positions, cells depleted of cherry-A3G became evident shortly after GFP-positive cells became detectable, with GFP-positive/mCherry-low cells predominating at later time points (Fig 4A and 4B). At intermediate time points, a significant number of GFP-expressing infected cells also retained unaltered mCherry-A3G expression (Fig 4A and 4B), consistent with the notion that there is a delay between expression of HIV-1 genes and removal of A3G from infected cells. As expected, and could be seen most clearly in HOS cells (Fig 4B), fewer GFP-positive cells retained mCherry-A3G when the GFP reporter gene was in the late position. In both cell lines, cells that expressed higher levels of the late GFP reporter were more likely to have depleted mCherry-A3G than cells that expressed lower levels of the late GFP reporter. Given that individual cells expressed progressively higher levels of late gene expression over time (Fig 2), this finding suggests that A3G depletion is more likely to have occurred in cells that have been expressing late genes for a longer period of time.

### Dynamics of A3G removal in individual HIV-1 infected cells

**Fig 5:**
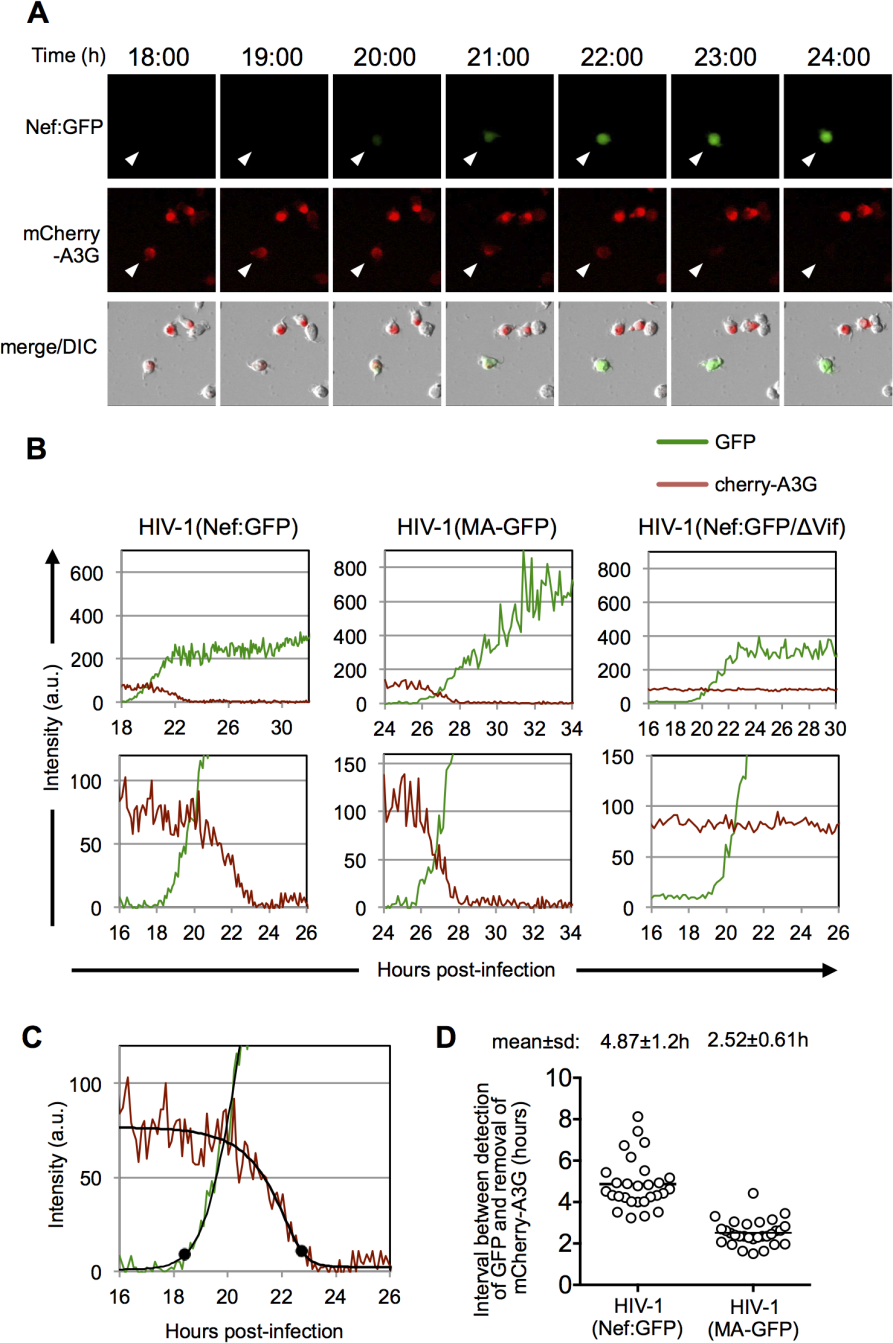
Dynamics of A3G down-regulation in individual HIV-1 infected cells. (A) Examples of images of individual MT-4 cells stably expressing an mCherry-A3G fusion protein and infected with an HIV-1(Nef:GFP). The time post-infection is indicated above the images. (B) Examples of quantitation of fluorescent intensities for individual MT-4 cells stably expressing an mCherry-A3G fusion protein infected with HIV-1(Nef:GFP) and HIV-1(MA-GFP) reporter viruses. An example of a cell infected with HIV-1(Nef:GFP) lacking a functional Vif protein is also shown Intensity units are 12-bit grey-level values, from 0–4095. Upper panels show data from entire experiments, lower panels show a magnified portion of the data around the time that HIV-1 gene expression became detectable. (C) Example of a 5-parameter logistic curve fit to the raw data and time points at which HIV-1 gene expression is detected and A3G is removed, defined here as the first point at which the GFP fit-curve rises 7 intensity units above its lower bound and the mCherry fit curve falls to 7 intensity units above its lower bound. The black dots indicate the identified time points for these events. (D) Time interval between the detection of HIV-1 gene expression, and removal of mcherry-A3G as defined in (C) for 28 individual HIV-1(Nef:GFP) infected cells and 29 individual HIV-1(MA-GFP) infected cells. Fluorescence data for each of these individual cells is shown in S4 and S5 Figs.

We next used the single-cell microscopy-based assay to measure mCherry-A3G levels in individual MT4 cells infected with HIV-1(Nef:GFP) or HIV-1(MA-GFP) carrying a GFP reporter in either early or late gene positions (Fig 5). In the presence of Vif, mCherry-A3G expression was visibly and progressively extinguished in infected cells as, or shortly after, GFP expression was detected (Fig 5A and 5B and S5–S8 Movies). Conversely, in the absence of Vif, mCherry-A3G expression levels remained stable over the course of infection (Fig 5B). To quantify the timing of A3G removal, we used the aforementioned MATLAB script to fit curves to the GFP and mCherry-A3G measurements for 28 cells infected with HIV-1(Nef:GFP) and 29 cells infected with HIV-1(MA-GFP) (Figs 5C and S4 and S5). Thereafter, we identified the time points at which GFP expression rose above background levels and mCherry expression dropped to background levels and calculated the time interval between them (Fig 5C). The mean±s.d. interval between the initiation of early gene expression to the completion of cherry-A3G removal was 4.9±1.2h, while the mean±s.d. interval between the initiation of late gene expression to the completion of cherry-A3G down-regulation was 2.5±0.6h (Fig 5D). Notably, the difference between these values, 2.4h, is in reasonable agreement with the value of 2.8h calculated for the interval between early and late gene expression using single-cell microscopy with dual-reporter viruses (Fig 2). Fluctuations in the levels of mCherry-A3G fluorescence, and heterogeneity in the trajectories of the best-fit curves made it difficult to assign time points at which A3G removal commenced. However, visual inspection of the fluorescence measurements in many individual cells (S4 and S5 Figs) suggested that A3G levels began to decline approximately coincident with the detection of late gene expression, thus A3G removal occurred over a period of ~2.5h in a typical cell.

### An approximate overall timeline for a single cycle of HIV-1 replication

**Fig 6:**
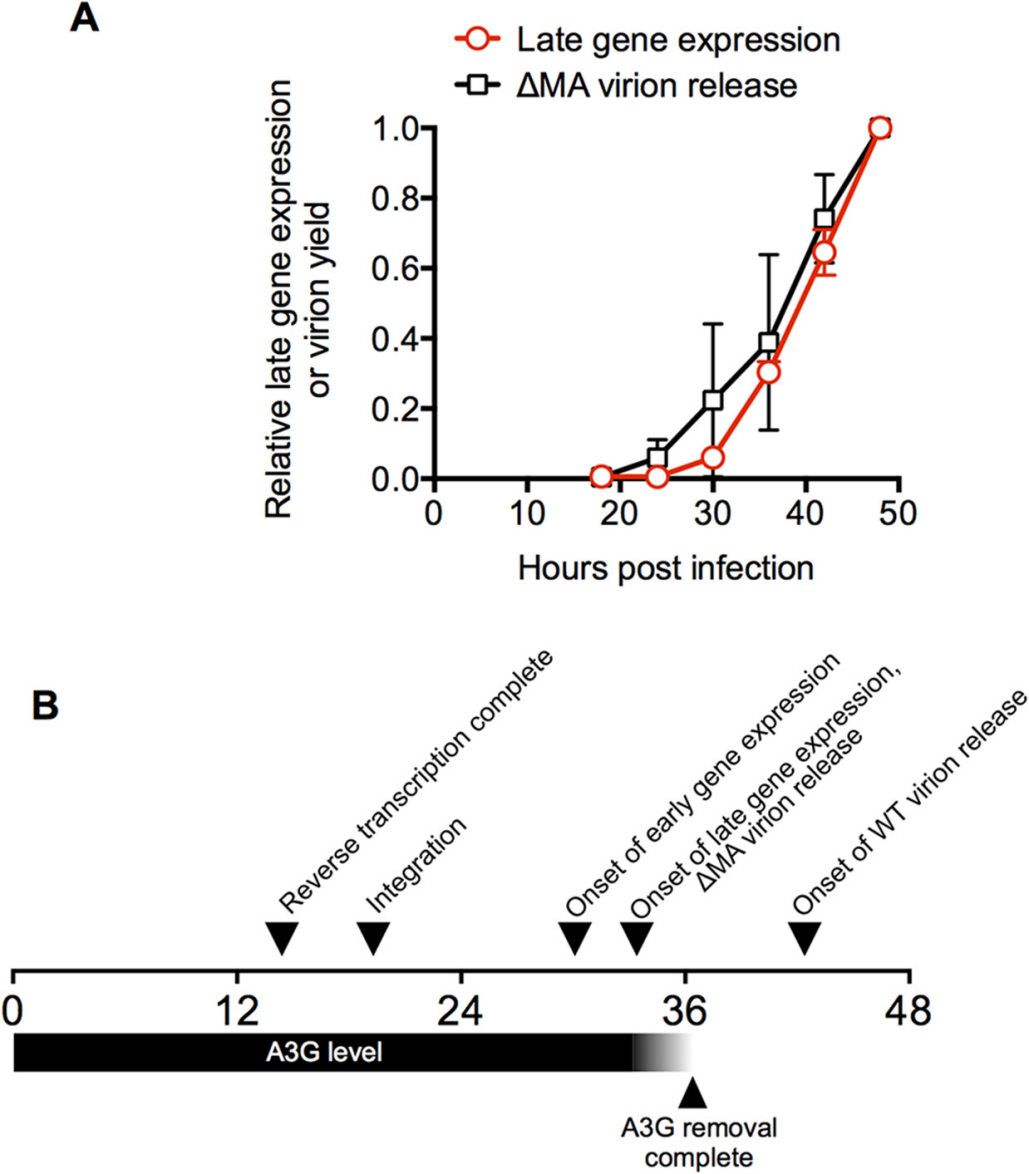
A time-line for a single cycle of HIV-1 replication in a typical cell. (A) Comparison of the dynamics of HIV-1 late gene expression and HIV-1(ΔMA) virion release in populations of cells. The mean ± sd (n = 4) of the proportion of cells expressing a late gene (Gag) reporter was calculated from the data presented as four individual curves in Fig 1C and plotted along with the level of virion release exhibited by HIV-1(ΔMA) infected cells (same data as in Fig 3C). (B) A time line for HIV infection in a typical MT4 cell. The horizontal scale indicates the time (in hours) after exposure to virus. Times for the completion of reverse transcription and integration, early and late gene expression are depicted as the 50% values for cell populations, as described in Fig 1. The timing for the completion of A3G removal is depicted relative to the detection of late gene expression, as determined in Fig 5. The interval between late gene expression and onset of WT virion release is depicted as the interval between initial detection of late gene or Gag expression in cells (which occurred at approximately the same time) and the initial detection of virion-associated CA protein in the culture supernatant.

Together, these experiments allow the generation of an approximate time-line of events in HIV-1 replication in a typical MT4 cell (Fig 6). Reverse transcription and integration are completed at 14.4h and 19.3h after infection in 50% of cells, while early and late gene expression are detected by FACS in 50% of cells at 30.1h and 33.4h hours post-infection, respectively. The 3.4h interval between the detection of early and late gene expression measured by FACS is in reasonable agreement with the interval (2.8h) measured using single live cell microscopy.

It is somewhat more challenging to relate virion assembly/release measurements to parameters that can be determined for individual cells, because the measured amount of released virions by populations of cells is governed by several parameters including: (i) the number of cells expressing Gag (i) the level at which they are expressing Gag and (iii) the rate at which virions are assembled and released. Both (i) and (ii) increase with time, given the asynchrony associated with HIV-1 replication, and (iii) also increases with time in the case of the WT virus given the cooperative nature of HIV-1 Gag assembly [42]. Nevertheless, the western blot analysis of HIV-1 Gag expression and particle release enabled some conclusions. First, Gag expression in cells was initially detected by western blot analysis at ~18h after infection (Fig 3). Importantly, this Gag expression was detected at about the same time as a small population of cells (a few percent) express detectable levels of the late gene reporter—note that the late gene reporter is embedded in the Gag protein and is, in fact, a surrogate measurement of Gag expression (Fig 1). Thus, Gag (or MA-GFP) detected at 18h represents expression by a small ‘leading’ cohort of cells in the asynchronously infected population that were the first to begin to express Gag. Second, in the case of the ΔMA virus, initial detection of extracellular virions occurred simultaneously with detection of cell-associated Gag and late gene reporter expression (Figs 1 and 3). Third, graphs describing extracellular virion accumulation for the ΔMA virus with time and the fraction of cells expressing the late gene (Gag) reporter exhibit similar trajectories (Fig 6A). Together, these finding suggest that, in the case of the ΔMA virus, there is no measurable delay between late gene (Gag) expression and virion assembly/release. Thus, if we make the assumption that a typical cell in the population is able to support virion assembly with similar kinetics to the ‘leading’ cohort of cells, then a typical ΔMA virus-infected cell would begin to release virions at 33.4h after infection (the same time as it begins to express late genes, including Gag). Conversely, WT virus infected cells generate extracellular virions only about 6–12h after detection of late reporter or Gag expression (Fig 3), leading to the conclusion that a typical WT virus infected cell would begin to generate extracellular virions at about 40 to 46h after infection.

Notably, the interval of about 2.8h between the onset of late gene expression and the completion of cherry-A3G removal (Fig 5) would place cherry-A3G removal between the times at which ΔMA and WT virions would be released. Thus, the delay in virion assembly imposed by MA results in the generation of virions only after A3G has been removed from the cell.

## Discussion

The relative paucity of information on the timing of the individual steps during the post-integration stages of the HIV-1 life cycle prompted us to develop approaches for measuring the progression of these processes. Specifically, we used viruses encoding fluorescent proteins that report levels of early and late (Gag) gene expression, coupled with flow cytometry, quantitative live cell microscopy, and quantitative western blotting to measure gene expression, virion production and removal of A3G from infected cells.

Even though we synchronized infection by employing a spinoculation and washing protocol, there was a significant degree of asynchrony in infected MT4 cell cultures as the HIV-1 replication cycle proceeded. This asynchrony primarily manifested itself prior to or during reverse-transcription, as evidenced by the significant variation (>18h) in the time required for infection events to escape the effect of RT inhibitors. Additional variation in the overall length of the viral life cycle, introduced between reverse transcription and gene expression, appeared comparatively modest, as evidenced by the rather parallel nature of the curves describing RT inhibitor escape and detection of early and late gene expression. Nevertheless, inherent asynchrony introduced early in the HIV-1 replication cycle complicates the measurement of the dynamics of the late stage of the viral life cycle. Therefore, a key feature of our analysis was the measurement of parameters in individual cells wherever possible, and enumeration of the numbers of individual cells in a population in which the viral replication cycle had progressed beyond a point that could be measured. Using these approaches, estimates of the timing of events in a typical infected cell were generated, as well as an appreciation of how much these values vary from cell to cell.

Our data revealed a significant interval between integration and the detection of early gene expression (~11h), a smaller interval (~3h) between early and late gene expression, and a longer interval (~6–12h) between late gene expression and the release of virion particles. The overall replication cycle in a typical cell required ~42h but was subject to a large degree of variation (as much as 24h) among individual cells, even in this relatively homogeneous cell population.

A few caveats accompany this analysis. First, we used VSV-G to mediate viral entry, and so the precise timing of viral entry may differ from that mediated by HIV-1 Env. However, the timing of entry and other steps in the HIV-1 replication cycle is likely to be intrinsically variable according to the cell type in which it is measured [1–5, 7, 8, 10, 50], and determined in part by the levels of receptors and dNTPs. The kinetics of other processes in the viral replication cycle measured here may also be cell-type dependent, depending on the availability of cofactors required for their execution. We performed our experiments using a highly permissive T-cell line, MT4, in which some steps may be more rapid than in primary cells. Nevertheless, our analysis of early and late gene expression in primary cells stimulated in various ways indicated that MT4 cells reasonably, albeit imperfectly, represented the properties of authentic HIV-1 target cells. Thus, the values given herein should be regarded as estimates of the timing of each occurrence in a typical MT4 cell and subject to variation within and between cell populations. Additionally, the timing of some events is obviously influenced by the techniques used to detect them. For example our measurements of gene expression involved detection of the protein products of early and late transcripts. Our measurements did not determine whether HIV-1 transcription is initiated immediately following integration (and 11h is required for HIV-1 mRNAs to be spliced and exported, and their translation product to reach a detection threshold) or whether there is a biologically determined delay between integration and transcription (perhaps, for example, governed by the cell cycle). Finally, our measurements of the removal of APOBEC3G make the assumption that the mCherry-fused protein is degraded with similar kinetics to that of the endogenous unfused protein.

Nevertheless, the relative timing of late events in the HIV-1 replication described herein is likely to be broadly correct. For example, the interval between early and late gene expression of about 3h was quite similar when measured in different ways: (i) using flow cytometric analysis of populations of cells expressing two different fluorophores as early and late genes, (ii) using microscopic analysis of individual cells expressing two different fluorophores as early and late genes (iii) using microscopic analysis of individual cells expressing a single fluorophore as an early or late gene and measuring the timing of its expression relative to the removal of cherry-A3G.

An unexpected finding was that early gene expression increased rapidly for several hours and then plateaued or increased at a slower rate thereafter, while late gene expression (Gag with an embedded fluorophore) continued to increase throughout the course of an ~48h experiment. This finding may reflect a degree of autoregulation of early versus late HIV-1 gene expression. Perhaps accumulated of Rev protein promotes diversion of incompletely spliced HIV-1 transcripts to the cytoplasm, limiting the number of nascent of transcripts that are available to be spliced for expression of early genes. A possible caveat is that the early reporter is expressed in an authentic unfused fluorescent protein, while the late reporter is a fluorescent protein embedded in Gag. While differential protein stability may affect accumulation of the two reporters, Gag is significantly less stable than unfused fluorescent proteins [51–53], and therefore differential protein stability cannot account for the elevated expression of late over early reporter genes at later time points. Moreover, one previous report has also suggested that the ratio of late:early HIV-1 mRNAs increases with time of infection [25].

A key conclusion of our studies is that, in the absence of the MA globular head, HIV-1 virion assembly proceeds promptly after late gene-expression. However, in the context of an intact Gag protein, virion egress is delayed by ~6–12h. Our own and others’ previous findings indicate that the MA globular head inhibits membrane binding, particularly at low Gag expression levels [39–42, 54]. Once the formation of an individual virion has been initiated at the plasma membrane, the assembly process itself occurs in minutes [55]. Thus, binding to the plasma membrane is likely a rate-limiting step in HIV-1 assembly and the MA globular head therefore causes a significant prolongation of the HIV-1 replication cycle. Two properties of MA appear to underlie its ability to retard Gag-membrane binding. First, when Gag is first expressed in the cytoplasm, it appears to be largely monomeric [56], a state in which the N-terminal myristate in sequestered in a hydrophobic pocket in the MA globular head [36]. Second, lysine residues in the N-terminal ~30 amino acids of the MA globular head that contribute to membrane binding also bind to particular tRNAs, specifically in the cytoplasm [33]. Thus, the two major signals in MA that drive Gag membrane binding are at least partly occluded when Gag is present in the cytoplasm. This autoinhibition of assembly caused by the MA globular head appears to be very significant in the context of a single cycle of HIV-1 replication, causing both a delay in assembly (about 6–12h) and an overall decrease in virion yield. Previous pulse-chase analyses of HIV-1 assembly have indicated that virions are released from the cell over a time period of 2–8h following the synthesis of WT Gag [52, 53, 57], thus demonstrating a significant time interval between WT Gag synthesis and its appearance in virions. While the values obtained using pulse chase analyses might appear to indicate that WT Gag assembles into virions more rapidly than do our experiments, it is important to note that these earlier studies were done under conditions where Gag proteins had already accumulated in transfected cells. Our analysis was done under conditions of a single infection cycle, in which the level of Gag expression increases with time. Because the rate of virion assembly increases with Gag expression levels, the descriptions of the kinetics of particle generation from the two different approaches are entirely compatible with each other.

The existence of antiviral proteins in infected cells may provide one impetus for this apparently programmed delay in HIV-1 egress. Molecules that inhibit the release of HIV-1 particles (CD4 and tetherin) or effectively poison progeny virions (A3G and other APOBEC proteins) are often present constitutively at significant levels in HIV-1-infected cells [45, 58, 59]. To accomplish the removal of these inhibitory proteins, HIV-1 employs accessory proteins (Nef, Vif and Vpu) that deplete or remove them from their sites of action. Our analysis shows that one consequence of the MA-imposed inhibition of HIV-1 assembly is to delay particle genesis to a time where A3G has been removed from the cell. The removal of other inhibitory molecules is likely to require similar delays in the replication cycle in order to be completed prior to virion production.

Other processes in HIV-1 replication might further impose a requirement for a delay in viral assembly. In order to generate infectious virions, Gag, Gag-Pol, Env and the viral genome must co-assemble at the plasma membrane. The dynamics of the accumulation of Gag-Pol and the viral genome might be reasonably assumed to be similar, given that the same RNA species serves as both the viral genome and the mRNA for Gag and Gag-Pol. However, given the complex modifications and trafficking that govern the localization of the HIV-1 Env protein, it is possible that virion assembly is delayed to enable the accumulation of functional Env spikes at the cell surface.

Two features of HIV-1 replication are emphasized by our findings: (i) there is significant heterogeneity in the length of the HIV-1 replication cycle, even in relatively homogenous MT4 cells, (ii) a typical infected cell generates new virions for only a few hours at the end of a 48h observation period. Notably, a typical infected T-cell *in vivo* lives for only ~48h [13]. If we assume that the length of HIV-1 replication cycle in T-cells *in vivo* is not significantly shorter than in highly permissive MT4 cells, then many infected cells generate virions for only a few hours at the end of their life. Therefore, interventions or cytotoxic immune responses that have only a small effect on the lifespan of infected cells might have a large effect on overall viral yield in a single cycle and the overall clinical course in an infected individual.

## Materials and Methods

### Cell culture

293T (ATCC #: CRL-11268) and HOS (ATCC #: CRL-1543) cells were obtained from ATCC and grown in DMEM supplemented with 10% fetal bovine serum. MT-4 cells, obtained from the NIH AIDS Research and Reference Reagent Program, were cultured in RPMI supplemented with 10% fetal bovine serum. Derivatives of HOS and MT-4 cells stably expressing cherry-A3G (cherry-A3G) were generated by transduction with a retroviral vector (pBMN-IRES-Blasti), that allowed expression of the gene of interest and Blasticidin resistance gene from the same transcript separated by an IRES, and were grown in medium supplemented with 10μg/ml Blasticidin. Stocks of the pBMN-cherry-A3G-IRES-Blasti retroviral vector were generated by co-transfection of the vector plasmid with plasmids expressing MLV Gag-Pol, VSV-G and HIV-1 Vif to block A3G-mediated effects on retroviral transduction. Peripheral blood mononuclear cells were isolated from blood using Ficoll-paque gradient centrifugation and CD4+ T-cells were isolated by negative selection (RosetteSep Human CD4+ T Cells Enrichment Cocktail, StemCell Technologies). Cells were activated with phytohemagglutinin (PHA-P, Sigma, 5μg/ml) or anti-CD3/CD28 beads (Dynabeads Human T-Activator CD3/CD28, Gibco) for 48h, and then grown in RPMI with 10% fetal bovine serum in the presence of IL-2 (20 or 30 U/ml) for a further 2 to 8 days before use. For some experiments (S1H and S1I Fig), CD4+ T-cells were stimulated with CD3/CD28 Dynabeads 6 days after initial stimulation with phytohemagglutinin, 48h h before infection.

### Viruses

The HIV-1 reporter viruses HIV-1(Nef:GFP), HIV-1(MA-GFP), HIV-1(MA-GFP/Nef:cherry), HIV-1(MA-cherry/Nef:GFP) were constructed using overlap PCR to insert GFP or cherryFP in either the Nef or Gag positions within the HIV-1 proviral clone HIV-1_NL4-3_ (dINdRTdEnv). The HIV-1_NL4-3_ (dINdRTdEnv) carries inactivating mutations in both the IN and RT open reading frames and a deletion in Env, rendering it replication-incompetent [55]. Reporters in the Nef position were inserted with the start codon of the fluorescent protein positioned identically to the native start codon of Nef. Reporters embedded in Gag were inserted in the stalk region of MA, immediately N-terminal to the protease cleavage site at the MA-CA junction, as previously described [55]. The protease site was duplicated immediately N-terminal to the fluorescent protein. For experiments in which virus particle yield was measured, an HIV-1 proviral clone NHG carrying GFP in the Nef position [60] was used. NHG was modified by the addition of a stop codon at nucleotide 2133 of the *env* gene removing the 142 C-terminal residues of the cytoplasmic tail of the Env protein, and by replacement of the V3 encoding region of *env* (nucleotides 915–975) with the V3 region from the R5-tropic proviral clone YU2. This proviral plasmid served as the backbone for the construction of (referred to as HIV-1 (ΔMA)) by the deletion of sequences encoding amino acids 7–110 of the MA protein. The presence of the cytoplasmic tail-deleted R5 tropic Env protein allowed the demonstration that the manipulations allowed the demonstration that the HIV-1 (ΔMA) virus was infectious, but was limited to a single cycle of infection in MT4 cells, which lack CCR5. Because the infectiousness of the HIV-1 (ΔMA) virus was significantly reduced compared to the WT virus we limited our analyses to production of physical particles. All DNA manipulations were performed using overlap-extension PCR and standard molecular biology techniques.

### Transfections and infections

All viral stocks used in these studies were pseudotyped with VSV-G and produced by transfection of semiconfluent 10cm plates of 293T cells with 10ug of proviral plasmid and 1ug of VSV-G expression vector, using polyethyleneimine (PEI; PolySciences) at a DNA:PEI ratio of 1:4. Single-cycle reporter viruses that carried inactivating mutations were produced by co-transfection of the proviral plasmid, an HIV-1_NL4-3_ GagPol-expression plasmid and a VSV-G expression plasmid, at a ratio of 5:5:1. Viral supernatants were filtered through a 0.22μm filter and stored in aliquots at -80°C. All viruses carried at least one fluorescent reporter gene, and viral titers were determined by infection of MT-4 cells with serially diluted viral stocks for 48 hours, and flow cytometry to measure the number of infected cells.

### Analysis of a single cycle of HIV-1 infection

All infections employed a synchronized infection protocol. Single-cycle virus containing supernatant was added to cells in 6-well or 12-well dishes in the presence of 5μg/ml polybrene. Cells were centrifuged at 350g, 15°C for 40 min, incubated at 37°C for 90 min (or 120 min for experiments where virion yield was determined) and then washed three times and fed with fresh medium. At various time points thereafter, cells were collected (following trypsinization for adherent cells) and fixed in a 2% final concentration of paraformaldehyde. Flow cytometry was performed on a CyFlow Space flow cytometer (PARTEC) using a blue 488nm laser for the excitation of GFP and a yellow 561nm laser for excitation of mCherry. Samples were loaded using a Hypercyte Autosampler 96-well plate-sampling machine (Intellicyt). Results were analyzed using FlowJo flow cytometry analysis software (Treestar Inc.). Anti-retroviral drugs were used in the inhibitor time course experiments and as controls in the assembly assays. Elvitegravir, an integrase inhibitor was used at 400nM. Efavirenz, a reverse transcriptase inhibitor was used at 200nM.

### Measurement of reporter gene expression in single cells

Cell-Tak adhesive (BD Biosciences) coated glass-bottom dishes were prepared on the day of use by adsorption with sodium bicarbonate and NaOH. MT4 cells, infected at a sufficiently low MOI so that each infected cell contained a single provirus were dispensed into these plates after washing, centrifuged at 350g for 8 minutes and immediately transferred to the microscope incubator. Time-lapse microscopy was performed using a VivaView FL incubator microscope (Olympus). Images of infected MT-4 cells were captured every 2–5 minutes using GFP, RFP, and DIC filter sets. Preparation of movies and quantitation of microscope data was done using Metamorph software (Molecular Devices). For quantitation, regions were drawn closely around immobilized cells and the maximum fluorescence intensity within each region was logged in both the GFP and mCherry channels. Fluorescence in unoccupied regions proximal to each cell was also quantified to provide background subtraction values for each cell. The background-corrected fluorescence intensities were analyzed using a custom MATLAB script that fit each set of fluorescent intensity data to a 5-parameter sigmoid logistic function. The function was used to identify time points where the signal was 7 intensity units above the baseline level. (Images had a bit-depth of 12, i.e., an intensity range of 0–4095).

### Western blotting

For experiments where virion yield was determined, a portion of the cells collected at each time point was resuspended in SDS-PAGE loading buffer. Simultaneously collected culture supernatants were clarified by centrifugation at 1000 rpm for 5 min filtered (0.22 μm), layered over 20% sucrose in 1X PBS and centrifuged at 14,000 rpm in an Eppendorf 5417R microfuge for 2 hours at 4°C. Virion pellets were resuspended SDS-PAGE loading buffer. Cell and virion lysates were separated on 4–12% acrylamide gels (Novex) and proteins transferred to nitrocellulose membranes which were then probed with antibodies against HIV-1 Gag (183-H12-5C, NIH AIDS Research and Reference Reagent Program). The blots were then probed with anti-mouse IgG conjugated to IR Dye800 CW (LiCOR), scanned with a LiCOR Odyssey IR imager and quantified using Odyssey quantification software.

## Supporting Information

S1 Fig Dynamics of HIV-1 replication measured using dual reporter viruses in primary and immortalized cells. (A-E) Purified primary CD4+ T-cells (A-D) or unfractionated peripheral blood mononuclear cells (E) from donor 1 (A, C) donor 2 (B, D) or donor 3 (E) were stimulated with PHA (A,B,E) or CD3/CD28 beads (C,D) and infected with HIV-1(MA-cherry/Nef:GFP). Cells were harvested at the times indicated on the X-axis and the percentage of GFP-positive and mCherry positive cells plotted. (F, G) The same purified primary CD4+ T-cells from donor 1 and 2 that were used in (A and B) were cultured for 8 days in IL-2 following PHA stimulation prior to infection with HIV-1(MA-cherry/Nef:GFP). (H, I) Purified primary CD4+ T-cells from donor 4 (H) or donor 5 (I) were stimulated first with PHA and then x days later with antiCD3/CD28 beads prior to infection with HIV-1(MA-cherry/Nef:GFP). (J, K, L) MT4 cells (J,K) or HOS cells (L) were infected with HIV-1(MA-cherry/Nef:GFP) (J,L) or HIV-1(MA-GFP/Nef:cherry) (K). Cells were harvested at the times indicated on the X-axis and the percentage of GFP-positive and mCherry positive cells plotted.
(TIFF)

S2 Fig Early and late gene expression in HIV-1(MA-cherry/Nef:GFP) infected cells. Fluorescent intensity traces and fit-curves for the individual HIV-1(MA-cherry.Nef:GFP) infected cells used for quantitation in Fig 2E are shown. The number below each plot represents the calculated interval between the onset of early and late gene expression for each infected cell.
(TIFF)

S3 Fig Early and late gene expression in HIV-1(MA-GFP/Nef:cherry) infected cells. Fluorescent intensity traces and fit-curves for the individual HIV-1 HIV-1(MA-GFP/Nef:cherry) infected cells used for quantitation in Fig 2E are shown. The number below each plot represents the calculated interval between the onset of early and late gene expression for each infected cell.
(TIFF)

S4 Fig Removal of mCherry-A3G in individual HIV-1(Nef:GFP) infected cells. Fluorescent intensity traces and fit-curves for the individual HIV-1(Nef:GFP) infected MT4/mCherry-A3G cells used for quantitation in Fig 5D are shown. The number below each plot represents the calculated interval between the onset of early gene expression and the completion of A3G removal for each infected cell.
(TIFF)

S5 Fig Removal of mCherry-A3G in individual HIV-1(MA-GFP) infected cells. Fluorescent intensity traces and fit-curves for the individual HIV-1(MA-GFP) infected MT4/mCherry-A3G cells used for quantitation in Fig 5D are shown. The number below each plot represents the calculated interval between the onset of late gene expression and the completion of A3G removal for each infected cell.
(TIFF)

S1 Movie Example#1 of an individual MT4 cell infected with HIV-1(MA-Cherry/Nef:GFP). Images were acquired in the GFP (upper left) RFP (upper right) DIC (lower left) channels and overlaid fluorescent images are displayed (lower right).
(MOV)

S2 Movie Example#2 of an individual MT4 cell infected with HIV-1(MA-Cherry/Nef:GFP). Images were acquired in the GFP (upper left) RFP (upper right) DIC (lower left) channels and overlaid fluorescent images are displayed (lower right).
(MOV)

S3 Movie Example#1 of an individual MT4 cell infected with HIV-1(MA-GFP/Nef:cherry). Images were acquired in the GFP (upper left) RFP (upper right) DIC (lower left) channels and overlaid fluorescent images are displayed (lower right).
(MOV)

S4 Movie Example#2 of an individual MT4 cell infected with HIV-1(MA-GFP/Nef:cherry). Images were acquired in the GFP (upper left) RFP (upper right) DIC (lower left) channels and overlaid fluorescent images are displayed (lower right).
(MOV)

S5 Movie Example#1 of an individual MT4/mCherry-A3G cell infected with HIV-1(Nef:GFP). Images were acquired in the GFP (upper left) RFP (upper right) channels. Overlaid fluorescent images are displayed (lower right) and overlaid fluorescent+DIC images are displayed (lower left).
(MOV)

S6 Movie Example#2 of an individual MT4/mCherry-A3G cell infected with HIV-1(Nef:GFP). Images were acquired in the GFP (upper left) RFP (upper right) channels. Overlaid fluorescent images are displayed (lower right) and overlaid fluorescent+DIC images are displayed (lower left).
(MOV)

S7 Movie Example#1 of an individual MT4/mCherry-A3G cell infected with HIV-1(MA-GFP). Images were acquired in the GFP (upper left) RFP (upper right) channels. Overlaid fluorescent images are displayed (lower right) and overlaid fluorescent+DIC images are displayed (lower left).
(MOV)

S8 Movie Example#2 of an individual MT4/mCherry-A3G cell infected with HIV-1(MA-GFP). Images were acquired in the GFP (upper left) RFP (upper right) channels. Overlaid fluorescent images are displayed (lower right) and overlaid fluorescent+DIC images are displayed (lower left).
(MOV)
