# Supplementary figures and images for: Single-Cell and Single-Cycle Analysis of HIV-1 Replication
Source: PLoS Pathog. 2015 Jun 18;11(6):e1004961. doi: 10.1371/journal.ppat.1004961 (PMC4472667; doi:10.1371/journal.ppat.1004961)

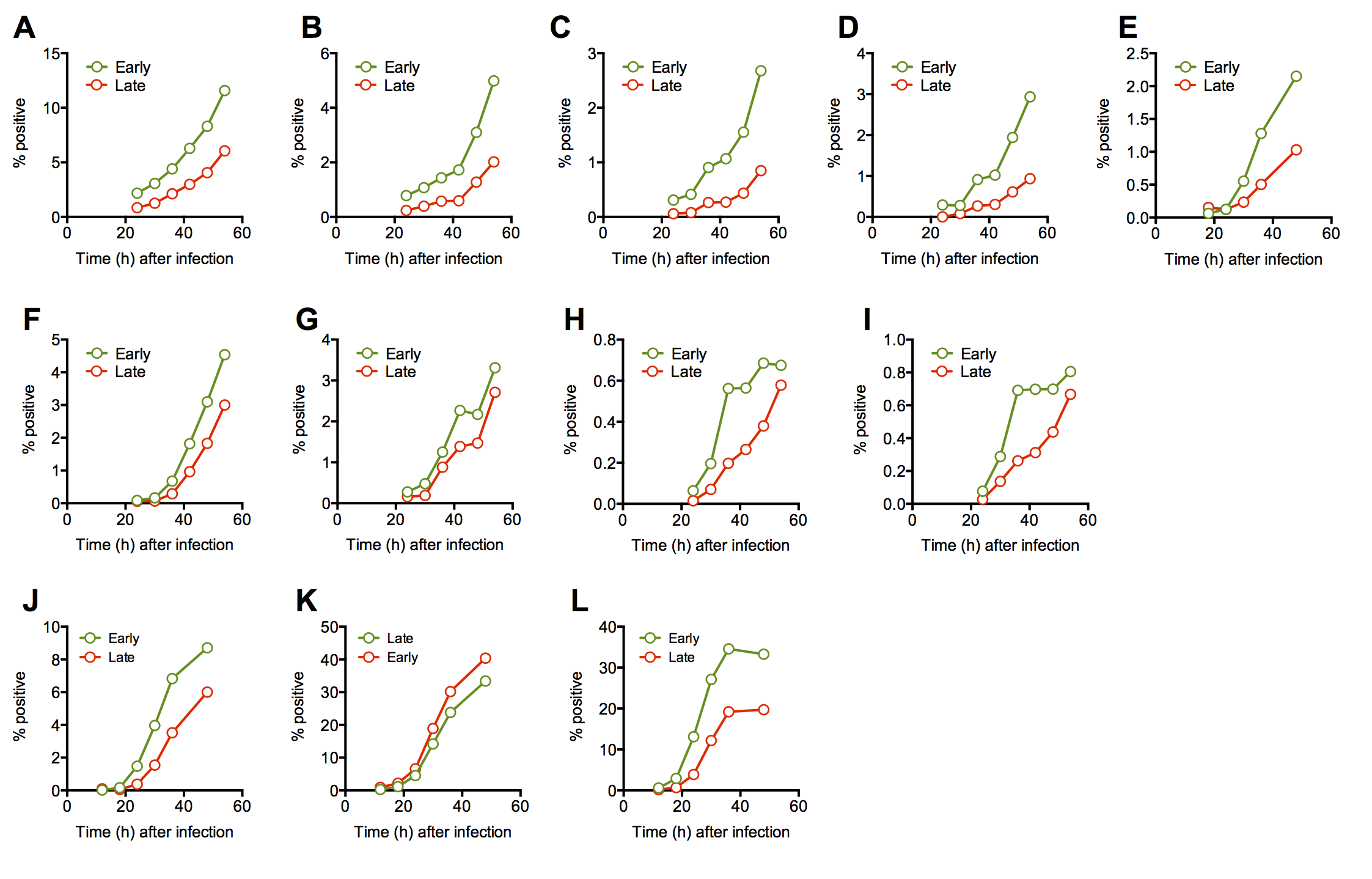

Supplement: S1 Fig — (A-E) Purified primary CD4+ T-cells (A-D) or unfractionated peripheral blood mononuclear cells (E) from donor 1 (A, C) donor 2 (B, D) or donor 3 (E) were stimulated with PHA (A,B,E) or CD3/CD28 beads (C,D) and infected with HIV-1(MA-cherry/Nef:GFP). Cells were harvested at the times indicated on the X-axis and the percentage of GFP-positive and mCherry positive cells plotted. (F, G) The same purified primary CD4+ T-cells from donor 1 and 2 that were used in (A and B) were cultured for 8 days in IL-2 following PHA stimulation prior to infection with HIV-1(MA-cherry/Nef:GFP). (H, I) Purified primary CD4+ T-cells from donor 4 (H) or donor 5 (I) were stimulated first with PHA and then x days later with antiCD3/CD28 beads prior to infection with HIV-1(MA-cherry/Nef:GFP). (J, K, L) MT4 cells (J,K) or HOS cells (L) were infected with HIV-1(MA-cherry/Nef:GFP) (J,L) or HIV-1(MA-GFP/Nef:cherry) (K). Cells were harvested at the times indicated on the X-axis and the percentage of GFP-positive and mCherry positive cells plotted. (TIFF) [file ppat.1004961.s001.tiff]

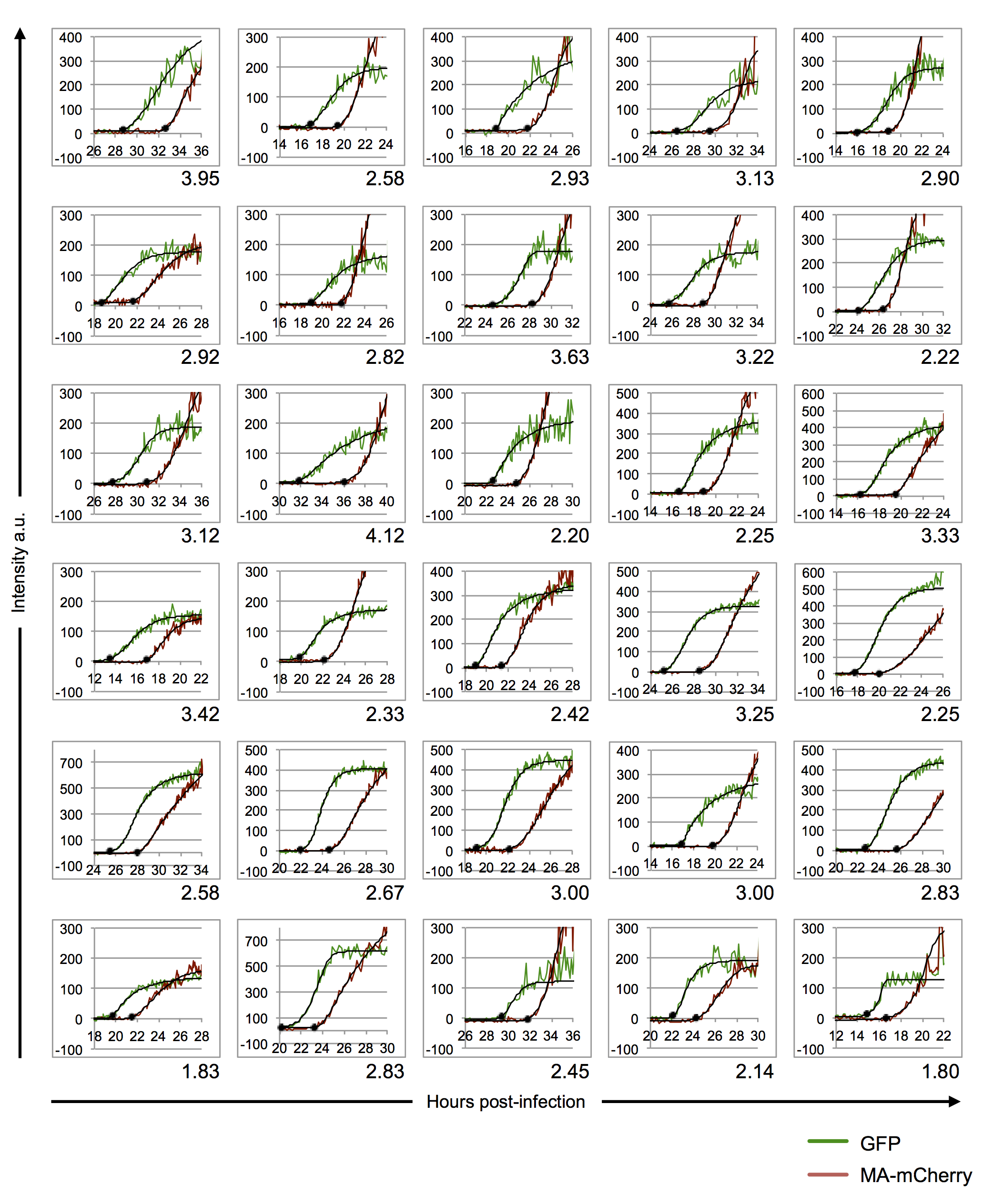

Supplement: S2 Fig — Fluorescent intensity traces and fit-curves for the individual HIV-1(MA-cherry.Nef:GFP) infected cells used for quantitation in Fig 2E are shown. The number below each plot represents the calculated interval between the onset of early and late gene expression for each infected cell. (TIFF) [file ppat.1004961.s002.tiff]

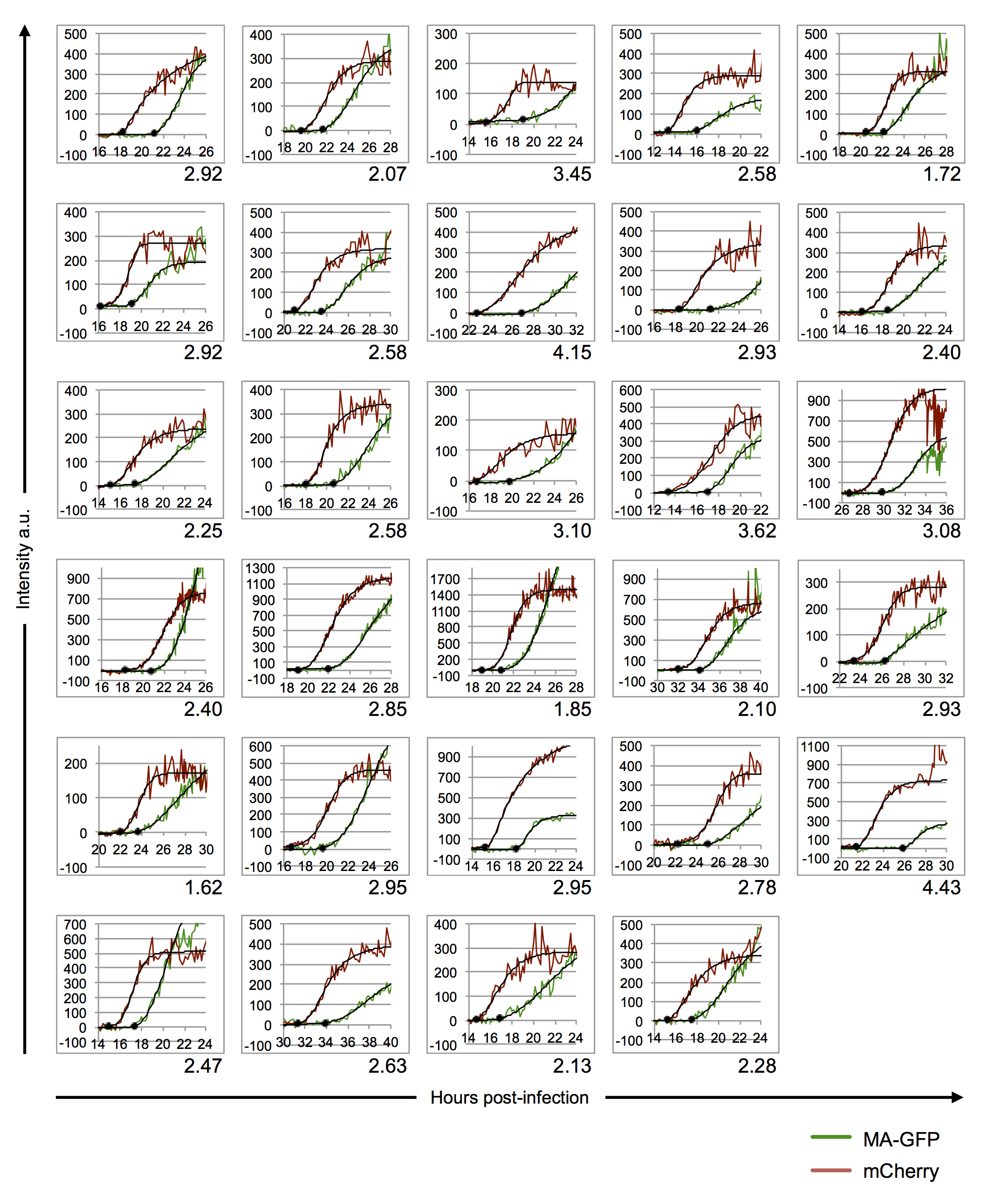

Supplement: S3 Fig — Fluorescent intensity traces and fit-curves for the individual HIV-1 HIV-1(MA-GFP/Nef:cherry) infected cells used for quantitation in Fig 2E are shown. The number below each plot represents the calculated interval between the onset of early and late gene expression for each infected cell. (TIFF) [file ppat.1004961.s003.tiff]

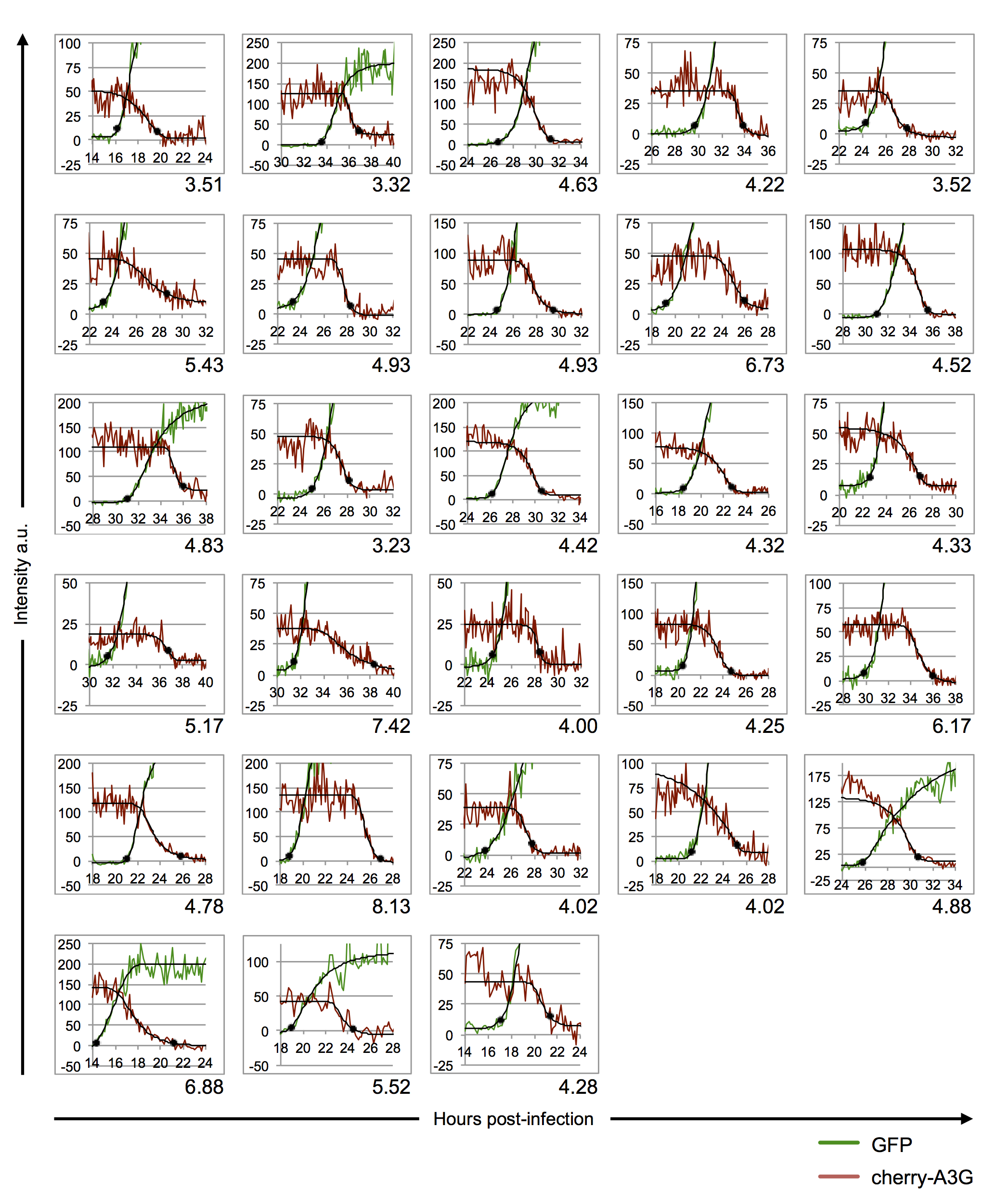

Supplement: S4 Fig — Fluorescent intensity traces and fit-curves for the individual HIV-1(Nef:GFP) infected MT4/mCherry-A3G cells used for quantitation in Fig 5D are shown. The number below each plot represents the calculated interval between the onset of early gene expression and the completion of A3G removal for each infected cell. (TIFF) [file ppat.1004961.s004.tiff]

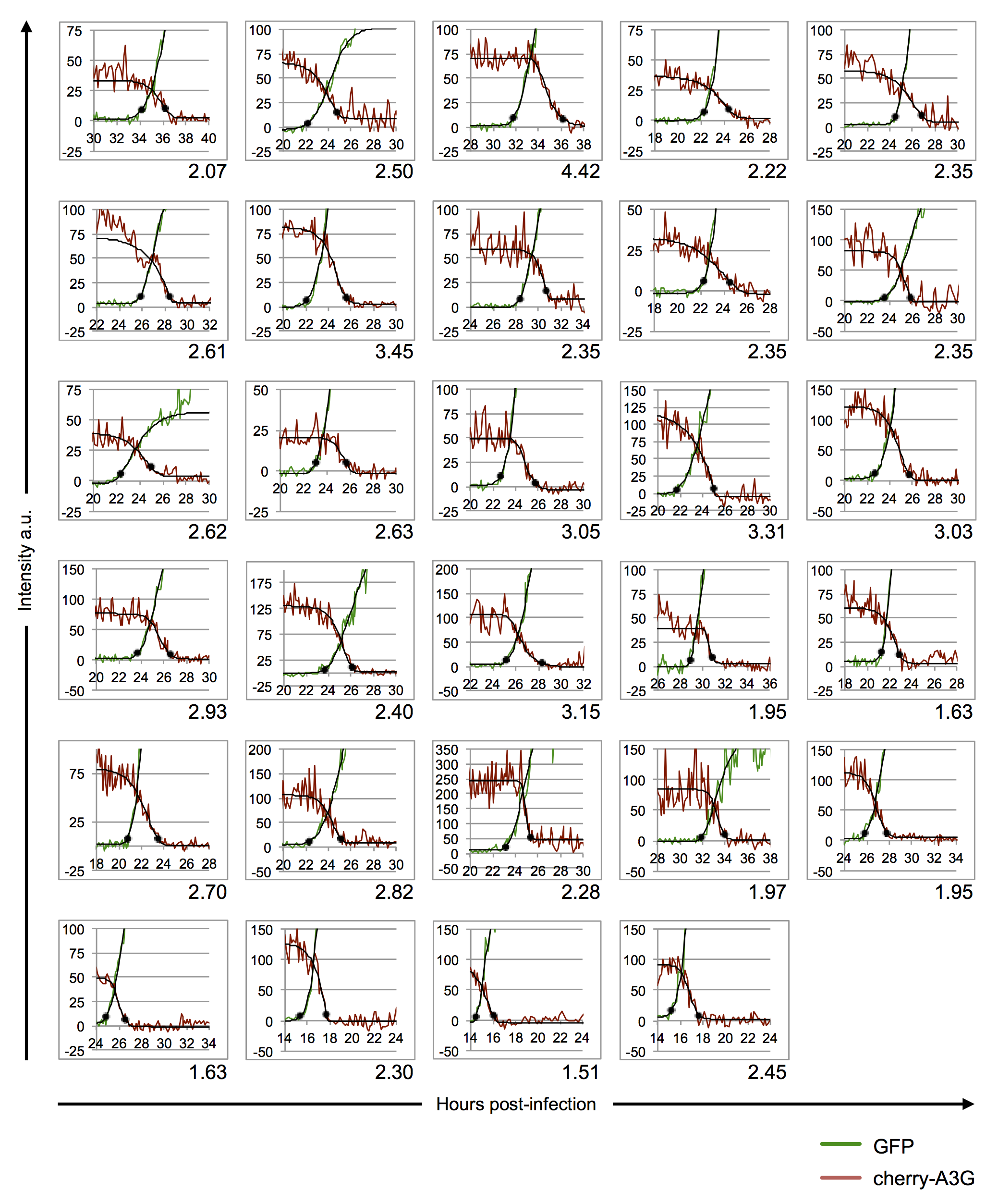

Supplement: S5 Fig — Fluorescent intensity traces and fit-curves for the individual HIV-1(MA-GFP) infected MT4/mCherry-A3G cells used for quantitation in Fig 5D are shown. The number below each plot represents the calculated interval between the onset of late gene expression and the completion of A3G removal for each infected cell. (TIFF) [file ppat.1004961.s005.tiff]
